# The critical window for host defense: macrophage trogocytosis eliminates *Echinococcus multilocularis* at the early establishment stage

**DOI:** 10.3389/fimmu.2026.1734332

**Published:** 2026-03-12

**Authors:** Ning Yang, Liang Li, Junlong Xue, Xue Zhang, Hongbin Zhang, Bowen Shi, Hui Liu, Jin Chu, Guodong Lü, Xiaojuan Bi, Renyong Lin

**Affiliations:** 1 State Key Laboratory of Pathogenesis, Prevention, and Treatment of Central Asian High Incidence Diseases, Clinical Medical Research Institute, The First Affiliated Hospital of Xinjiang Medical University, Urumqi, Xinjiang, China; 2 Key Laboratory of High Incidence Disease Research in Xingjiang (Xinjiang Medical University), Ministryof Education, Urumqi, Xinjiang, China

**Keywords:** *Echinococcus multilocularis*, host defense, macrophage, trogocytosis, complement system

## Abstract

**Introduction:**

The ability of *Echinococcus multilocularis (E.m) * to establish infection depends on evading host immune clearance during the early stages. This study investigated the mechanisms by which host macrophages eliminate *E.m* during this critical window.

**Methods:**

We utilized a mouse infection model with fluorescently labeled protoscoleces to observe early immune dynamics *in vivo*. Histopathological analysis was performed to characterize lesion phenotypes. The mechanism of parasite killing was further explored using *in vitro* co-culture experiments and scanning electron microscopy (SEM).

**Results:**

Rapid immune cell infiltration and parasite clearance in the liver were observed within 24 hours post-infection. Histopathological analysis revealed two distinct lesion phenotypes: "Progressive Lesions," characterized by a failure of macrophage infiltration and parasite transformation into vesicles, and "Regressive Lesions," marked by high macrophage density and complete parasite elimination. *In vitro* experiments demonstrated that macrophages mediated protoscolex killing through complement-dependent trogocytosis, a process requiring active serum components. SEM confirmed direct macrophage-parasite contact and trogocytosis as the primary mode of elimination.

**Discussion:**

These findings highlight the pivotal role of macrophage trogocytosis in early host defense against *E.m* infection and provide new insights into the mechanisms of innate immunity in parasitic clearance.

## 1 Introduction

*Echinococcus multilocularis* (*E.m*), the causative agent of alveolar echinococcosis (AE), is a highly pathogenic zoonotic parasite that primarily parasitizes the dogs, foxes, and rodents (1). In humans, the larval stage of *E.m* predominantly establishes in the liver, where it infiltrates the hepatic tissue in a tumor-like manner, leading to progressive liver damage, fibrosis, and potentially fatal complications if left untreated (2). Despite advances in chemotherapy and surgical interventions, recurrence and incomplete parasite clearance persist as major clinical challenges, particularly in endemic regions (3, 4). The establishment of chronic infection by this parasite critically depends on its ability to evade host immune responses at the early establishment stage of infection, thus determining subsequent disease outcomes.

The early establishment phase of *E.m* infection in the liver represents a critical period determining whether host immunity successfully eliminates the parasite or permits its persistence (5, 6). The hepatic immune microenvironment, particularly the activity of resident macrophages (Kupffer cells) and recruited myeloid cells, plays a decisive role in this process (7). While macrophages are known to engulf and destroy pathogens, how they specifically target the invasive larvae stage of *E.m* remains incompletely understood. Previous studies suggest that direct cellular contact and soluble immune factors, such as complement proteins, may contribute to parasite clearance (8–10). However, the critical factors determining macrophage efficacy in eradicating the parasites remain elusive. Uneliminated parasites can ultimately undergo metamorphosis into metacestode vesicles, facilitating long-term parasitism.

Although the recruitment of macrophages to the infection sites in *E.m* infection is well-documented (7, 11, 12), the specific effector mechanism that determine parasite fate—elimination or immune evasion—i poorly defined. In cases where early clearance fails, parasites undergo metamorphosis into metacestode vesicles within the liver, driving chronic inflammation, progressive fibrosis, and eventual organ dysfunction (13). Trogocytosis, the process by which immune cells “nibble” on fragments of target cells, has recently emerged as a key mechanism of anti-parasitic immunity (14). However, whether it serves as the primary mode of macrophage-mediated clearance of *E.m* and what key regulators govern this process remain unexplored. The aim of this study was to investigate the spatiotemporal dynamics of macrophage-parasite interactions at the early stage of infection as well as to determine whether macrophages clear *E.m* primarily through trogocytosis.

Our findings not only elucidate a key clearance mechanism but also suggest that modulating the complement pathway (e.g., with complement agonists) could be a therapeutic strategy to bolster macrophage trogocytosis and host resistance. Future work must carefully assess the potential risks of such immunomodulation, particularly the induction of excessive inflammation.

## 2 Materials and methods

### 2.1 Ethics statement

All mouse experiments were approved by the Institutional Animal Care and Use Committee as well as the Ethical Committee of the First Affiliated Hospital of Xinjiang Medical University (Approval No. K202110-18) and complied with ARRIVE guide for the care and use of laboratory animals. Mice were anesthetized with isoflurane during protoscolex injection and euthanized by cervical dislocation under deep anesthesia at designated timepoints.

### 2.2 Parasite materials and mice

Protoscoleces (PSCs) were isolated from intraperitoneal lesions in Mongolian gerbils as previously reported (15). The isolated PSCs were then carefully rinsed with phosphate-buffered saline (PBS) and counted microscopically. The viability of PSCs was evaluated by 0.1% methylene blue (G1300, Solarbio) staining, and only PSCs with ≥ 95% viability were used for subsequent experiments. Forty female C57BL/6J mice, aged 6–8 weeks, 18–20 g body weight were sourced from Vital River Laboratory Animal Technology Co., Beijing, China. Thirty-six mice were randomly divided into six groups: the sham operation group (Sham, n = 6) and five *E.m* infection model groups (1, 3, 5, 7, and 10 days post-infection (dpi), n = 6 each). Additional mice were used for preliminary fluorescence tracing experiments. All animals were maintained under standardized specific pathogen-free (SPF) conditions at 22 ± 2 °C with 50 ± 10% relative humidity and a 12 h light/dark cycle to maintain normal circadian rhythms.

### 2.3 Establishment of *E.m* infection models and sample collection

Mice in *E.m* infection groups were inoculated with PSCs via the hepatic portal vein, receiving 3000 PSCs diluted in 200 µL normal saline as previously described (15), while mice in the sham operation group were injected with equivalent amount of normal saline under anesthesia with isoflurane. *E.m*-infected mice were euthanized at 1, 3, 5, 7, and 10 dpi, while Sham group mice were sacrificed at 10 dpi. Liver tissues were collected and divided into two portions: one was immediately snap-frozen in liquid nitrogen for cryosectioning and subsequent fluorescence staining analyses, and the other was fixed in 4% paraformaldehyde for 72 h at 4 °C followed by paraffin embedding for histopathological examination.

### 2.4 Fluorescent labeling experiments

For *in vivo* tracing, PSCs were incubated with 5 μg/ml Dil (42364, Sigma-Aldrich) in RPMI-1640 at 37 °C for 30 min and then rinsed three times with PBS. For *in vitro* co-culture of dual fluorescent labeling, resident peritoneal macrophages (RPMs) were labeled with 5 μg/ml Dil and incubated with 5 μg/ml DiD (D7778, Invitrogen) labeled PSCs in RPMI-1640 at 37 °C for 30 min, then rinsed three times with PBS. Using 3D scanning to capture macrophages undergoing trogocytosis, with a step size of 3 μm. Image acquisition and analysis were performed using a laser confocal microscope (Nikon AX).

### 2.5 Histopathological and immunofluorescence analysis

Serial paraffin sections (4 μm thick) were stained with hematoxylin and eosin (H&E) and Sirius red following established protocols (16). Imaging was performed using light microscopy (DM3000, Leica, Germany), and Sirius red-stained sections were quantitatively analyzed with the Image-Pro Plus software (Version 6.0.0.260, Media Cybernetics, USA) based on positively stained areas. For immunofluorescence detection of macrophage-mediated *E.m* lesion clearance, frozen liver sections (5 μm thick) from each group were sequentially incubated with F4/80 primary antibody (70076, CST) targeting macrophages overnight at 4 °C, followed by AF-488-conjugated secondary antibody (4412, CST). Imaging and analysis were conducted using a laser scanning confocal microscope (Leica SP8).

### 2.6 *In vitro* RPMs-PSCs co-culture experiments

RPMs were chosen for this study because they are tissue-resident macrophages that functionally and phenotypically resemble liver Kupffer cells, the cells that first encounter the parasite during natural infection, and prepared as previously reported (17). Briefly, peritoneal lavage was performed on 6–8 weeks old C57BL/6 mice using 10 ml of ice-cold endotoxin-free PBS (without Ca^2+^/Mg^2+^). The lavage fluid was centrifuged (400×g, 10 min, 4 °C), and cells were purified by adherence (2 h, 37 °C/5% CO_2_ in RPMI-1640 medium supplemented with 10% FBS (Gibco)). The harvested cells were then cultured for 7 days in complete medium containing 40 ng/ml murine macrophage colony-stimulating factor (M-CSF, MedChemExpress) to induce differentiation into RPMs. For co-culture experiments, RPMs were co-cultured with PSCs at a ratio of 500:1 (RPMs: PSCs) for 72 h in RPMI-1640 for the Mø+PSCs group, 10% heat-inactivated mouse serum (HI-MS) (56 °C for 30 min) for the Mø+PSCs+HI-MS group, 10% normal mouse serum for the Mø+PSCs+MS group, and antibiotics (penicillin/streptomycin, 1:100) in all groups.

### 2.7 Scanning electron microscopy analysis

For SEM analysis, the co-cultured PSCs were fixed in 2.5% glutaraldehyde in 0.1 M sodium cacodylate buffer for 48 h at 4 °C, followed by several washes in the same buffer. Dehydration was performed through a graded ethanol series (30%, 50%, 70%, 90%, and 100%), and samples were then immersed in hexamethyldisilazane (HMDS) for 5 min, 1 h, and overnight to ensure complete drying. The dried specimens were sputter-coated with a 100 Å gold layer to enhance conductivity. Imaging was conducted using a JEOL JSM-6390 LV scanning electron microscope operated at 15 kV, and morphological changes in trogocytosis RPMs on PSCs were assessed.

### 2.8 Complement rescue experiment

To perform complement rescue experiments, RPMs and PSCs were co-cultured at a 500:1 ratio in serum-free RPMI-1640 medium containing penicillin/streptomycin (1:100) for 48 hours. The cultures were supplemented with recombinant C3 (1 µg/ml, CO3-M52H4, ACROBiosystems), C5 (1 µg/ml, CO5-M52H4, ACROBiosystems), or both proteins simultaneously (co-treatment group). As a control for potential cytotoxicity, PSCs were cultured alone under identical conditions with the respective recombinant complement proteins.

### 2.9 Statistical analysis

GraphPad Prism (Version 8.0.1, CA, USA) was used for statistical analyses. The normality of data residuals was determined by the Shapiro-Wilk test. Comparisons of continuous variables between two specific groups were performed using unpaired two-tailed Student’s t-test. Comparisons of means among multiple groups were conducted by one-way ANOVA followed by Tukey’s *post hoc* test for multiple comparisons. *P*-value < 0.05 was considered statistically significant.

## 3 Result

### 3.1 Fluorescent labeling enables efficient *in vivo* tracking of PSCs

**Figure 1:**
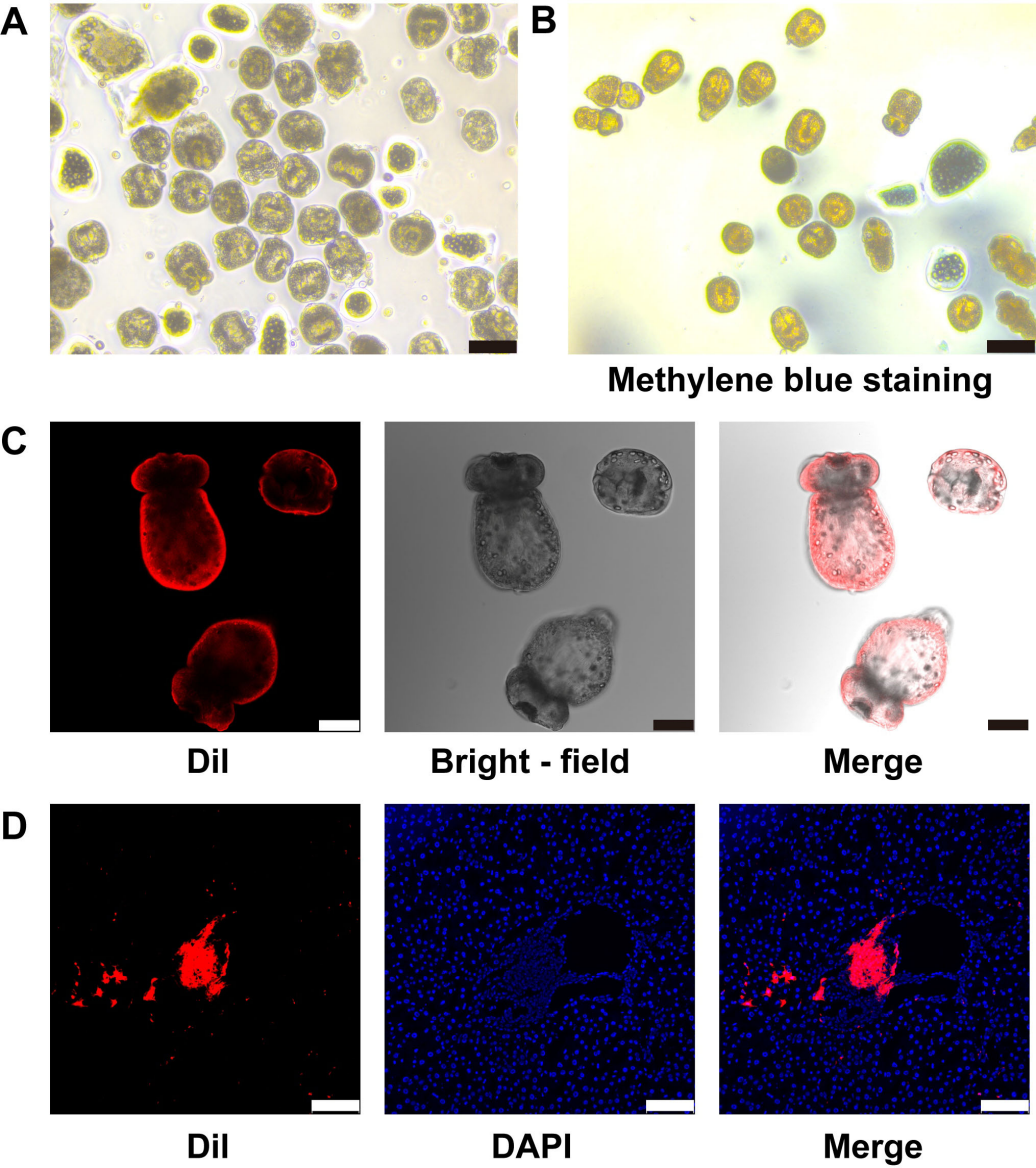
*In vitro* labeling and *in vivo* tracking of PSCs. **(A)** Representative image of *in vitro* culture of PSCs. Scale bar, 100 µm. **(B)** Viability assessment of PSCs using methylene blue staining. PSCs stained blue are dead. Scale bar, 100 µm. **(C)** Representative images of PSCs labeled with the fluorescent lipophilic dye Dil. Scale bar, 75 µm. **(D)** Representative images demonstrating the distribution of Dil-labeled PSCs (red fluorescence) in the mouse liver at 1 dpi with *E. multilocularis* infection. Blue fluorescence, nucleus. Scale bar, 100 µm. Data are representative of three independent experiments with similar results.

To explore the interactions between *E.m* and host immune cells, we established a mouse infection model by fluorescently labeling PSCs *in vitro* with Dil dye and injecting them into the portal vein. First, protoscolex viability was assessed using methylene blue staining. Only PSCs with viability ≥95% were subjected to subsequent fluorescent labeling (**Figures 1A, B**). *In vitro* labeling of PSCs with the lipophilic dye Dil showed successful staining mainly on the tegument, clearly outlining their morphology, while not affecting parasite viability. At 24 h post-infection, fluorescence analysis of liver cryosections from sacrificed mice revealed numerous Dil - labeled PSCs distributed around the portal vein areas and other vascular regions, consistent with the hematogenous liver invasion route, with some intact parasites still present (**Figures 1C, D**). These findings confirm that Dil labeling is an effective method for *in vivo* tracking of PSCs and a new tool for studying parasite-host interactions.

### 3.2 Hepatic pathology and host response at the early establishment stage of *E.m* infection

**Figure 2:**
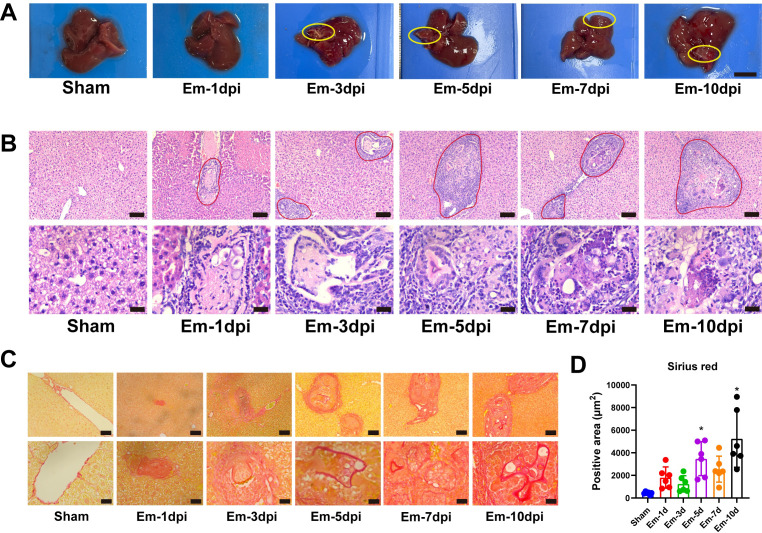
Histopathological changes of mouse liver at the early stage of *E.m* infection. **(A)** Representative gross images of mouse livers at different post-infection time points. Scale bars: 0.5 cm **(B)** H&E staining of liver sections at different post-infection time points. Scale bars: upper panels, 100 μm; lower panels, 25 μm. **(C)** Sirius red staining of liver sections at different post-infection time points. Scale bars: upper panels, 100 μm; lower panels, 25 μm. The area outlined in solid red line represents the lesion. **(D)** Quantification of Sirius red-positive areas in liver tissues. Data are presented as mean ± SD (n = 6 per group). Statistical analyses were performed using one-way ANOVA with Tukey’s *post hoc* test. *P* < 0.05 compared to the Sham group.

Successful parasite clearance at the early stage is critical for host resistance to *E.m* infection. To elucidate key pathological events at the early stage, particularly host immune responses, infected mice were sacrificed at 1, 3, 5, 7, and 10 dpi for histopathological analysis of livers. Gross liver examination revealed normal morphology in the Sham group, while the infected groups exhibited scattered punctate white lesions at all time points, though no distinct cyst formation was evident yet (**Figure 2A**). H&E staining revealed immune cell infiltration around hepatic lesions as early as 1 dpi, indicating that *E.m* infection rapidly triggers the host immune responses. Notably, intact PSCs were observed within some lesions at 1–3 dpi. However, significant structural alterations in PSCs occurred after 5 dpi. In some lesions, immune cells infiltrate the lesion, and fragmented PSC structures are observed, suggesting active immune-mediated damage. In parallel, within other lesions, PSCs underwent metamorphosis, initiating a structural transformation toward vesicle, leading to the expansion of the lesion. In addition, there are lesions that no longer contain visible protoscoleces but are surrounded by an inflammatory reaction and form granuloma-like structures (**Figure 2B**). Sirius red staining revealed progressive fibrosis within the areas of inflammatory cell infiltration surrounding lesions, with the fibrotic area expanding alongside lesion growth (**Figures 2C, D**). These results demonstrate that the host mounts an early immune response to *E.m* infection aimed at clearance. The ability of PSCs to resist immune-mediated clearance and successfully undergo metamorphosis into vesicles is crucial for establishing successful parasitism.

### 3.3 *In vivo* tracing demonstrates that macrophages participate in the early clearance of *E.m* infection

**Figure 3:**
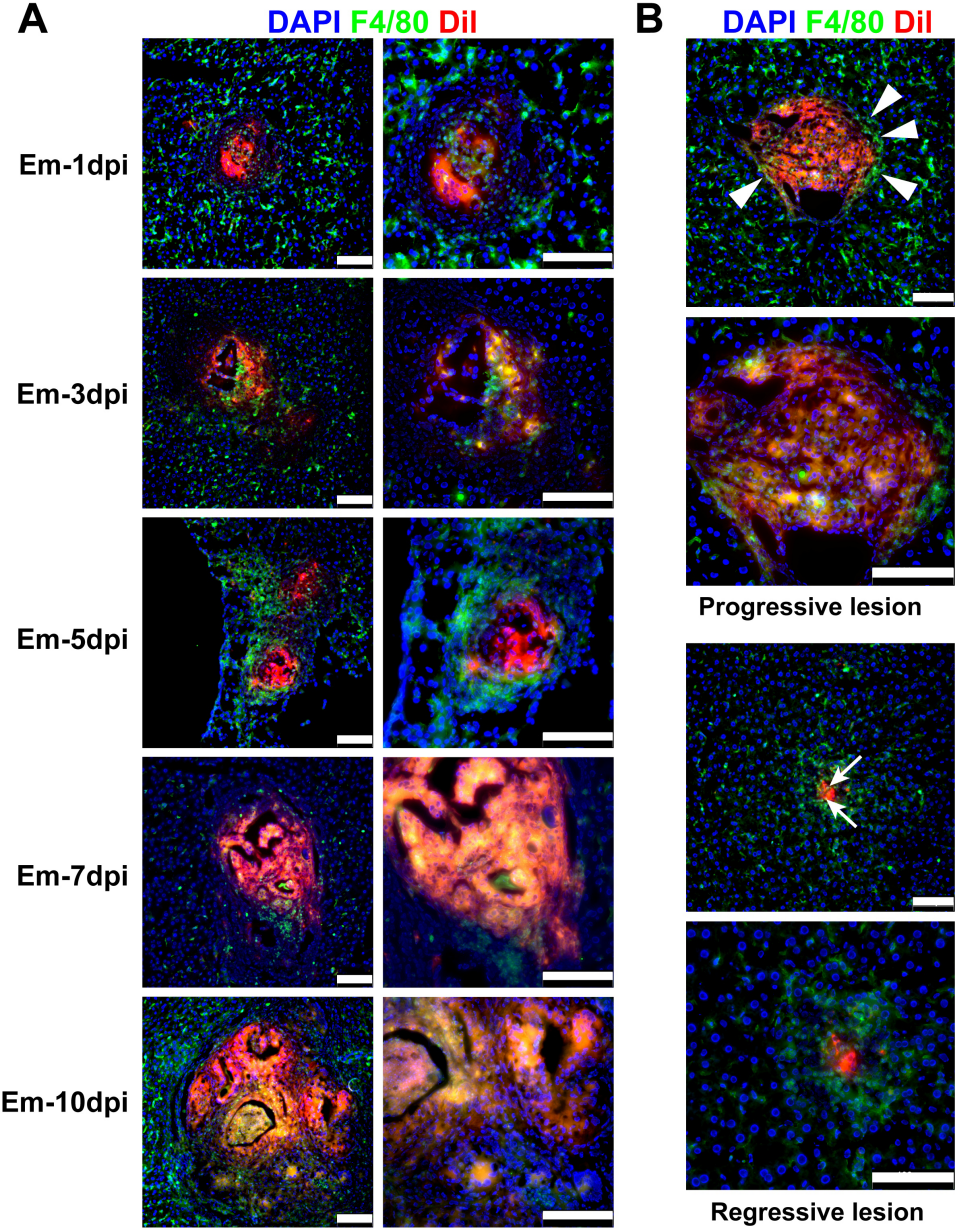
Spatial dynamics of macrophage infiltration dictate lesion fate in *E.m* infection. **(A)** Spatiotemporal infiltration of F4/80^+^ macrophages (green fluorescence) into *E.m* lesions (red fluorescence) at 1, 3, 5, 7, and 10 dpi. Scale bars, 100 μm. Right column (magnified views). Images are representative of results from 6 mice per group. **(B)** Representative images of different lesion phenotypes at 10 dpi. Upper panels: Progressive lesion showing peripheral macrophage localization (White arrowheads) around developing vesicular structures. Lower panels: Regressive lesion exhibiting core-infiltrating macrophages (White arrows) overrunning parasite foci. Scale bars, 100 μm. Blue fluorescence, nucleus.

Our previous studies have demonstrated that host clearance of *E.m* infection at the early stages primarily relies on innate immunity. As core effector cells of innate immunity, macrophages play a pivotal role in the anti-parasitic immune response at the early establishment stage of *E.m* infection (7). To investigate the potential mechanisms of macrophage clearance, we performed immunofluorescence staining of mouse liver tissue sections for the macrophage marker F4/80, combined with *in vivo* tracking of the PSCs at 1, 3, 5, 7, and 10 dpi. Analysis of fluorescence imaging revealed that macrophages infiltrated the core of the lesions at the early stage of *E.m* infection, establishing direct contact with parasites to facilitate clearance (**Figure 3A**). Interestingly, after detailed analysis of lesions with divergent developmental outcomes, we identified two distinct lesion phenotypes: clearance-resistant “Progressive Lesions” exhibited vesicular structure formation with macrophages localized peripherally around parasite masses, demonstrating failure to infiltrate lesion interiors. Conversely, the “Regressive Lesions” displayed significantly higher macrophage density, with complete overrunning of protoscolex-occupied regions. The parasite structures were largely completely eliminated, and these lesions subsequently developed into granulomas until they are completely repaired (**Figure 3B**). These findings indicate that effective macrophage infiltration into the core of the lesion is critical for host resistance to the *E.m* infection, and this resistance hinges on the ability of macrophages to establish direct contact with the parasites.

### 3.4 Direct macrophage-parasite contact is critical for clearing early *E.m* infection and preventing lesion progression

**Figure 4:**
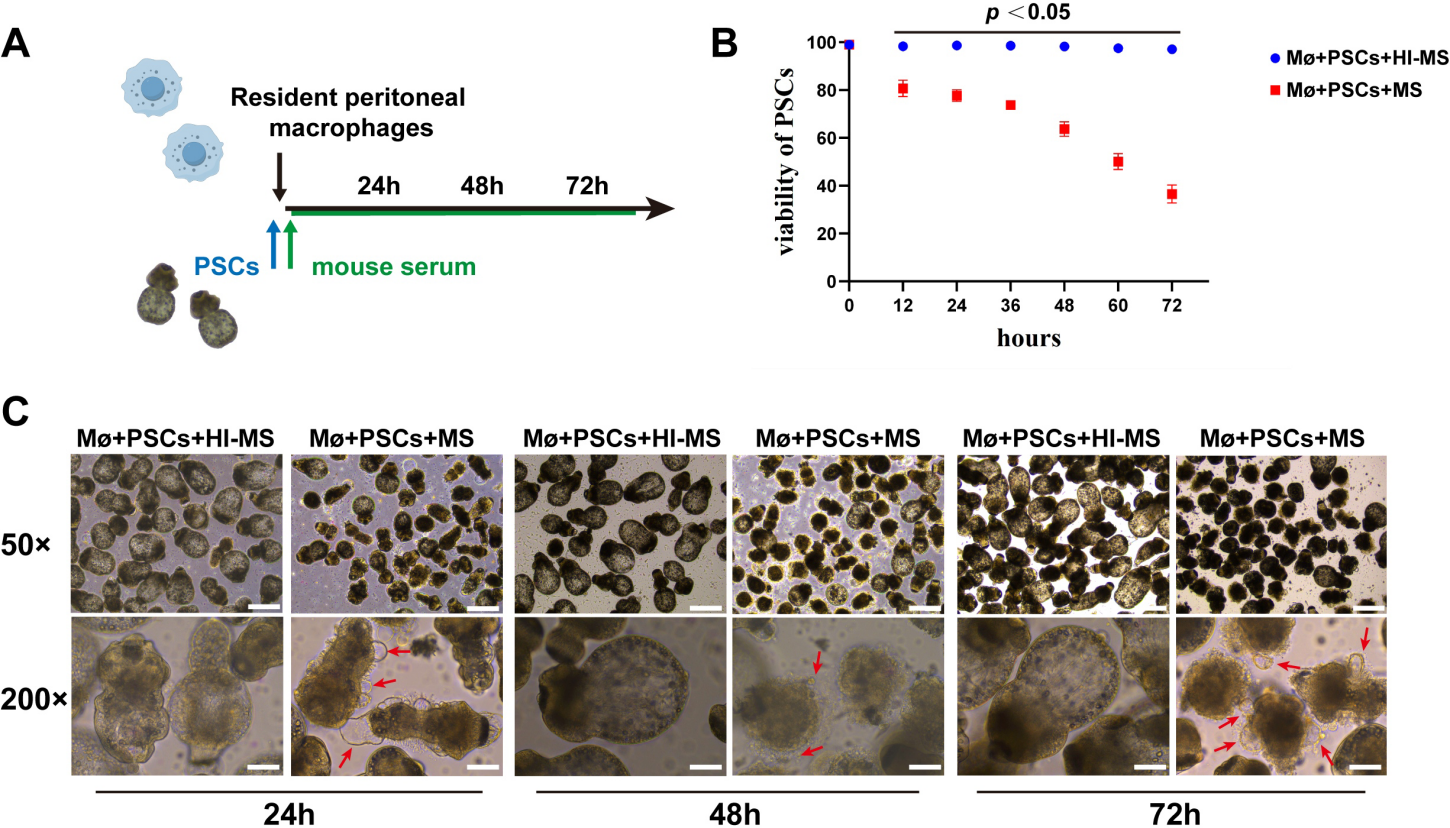
*In vitro* co-culture reveals components in mouse serum mediate the cytotoxic effects of macrophages on PSCs. **(A)** Schematic of experimental design: PSCs co-cultured with RPMs for 72 h supplemented with heat-inactivated (HI-MS) or active mouse serum (MS). **(B)** Viability kinetics of PSCs measured by methylene blue staining at 12 h intervals. Statistical analyses were performed using unpaired two-tailed Student’s t-test. *P* < 0.05 compared to the HI-MS group at the same time point (mean ± SD, n = 3). **(C)** Morphological changes in PSCs under different conditions during 72 h observation. Red arrows indicate vacuolar changes and dead PSCs. HI-MS group: Minimal contacts, preserved PSC morphology. MS group: Progressive PSC degeneration with macrophage contacts. Macrophages engaged in contact-dependent killing are indicated by red arrows. Scale bars: upper panels, 100 μm; lower panels, 50 μm. Data are representative of three independent experiments.

**Figure 5:**
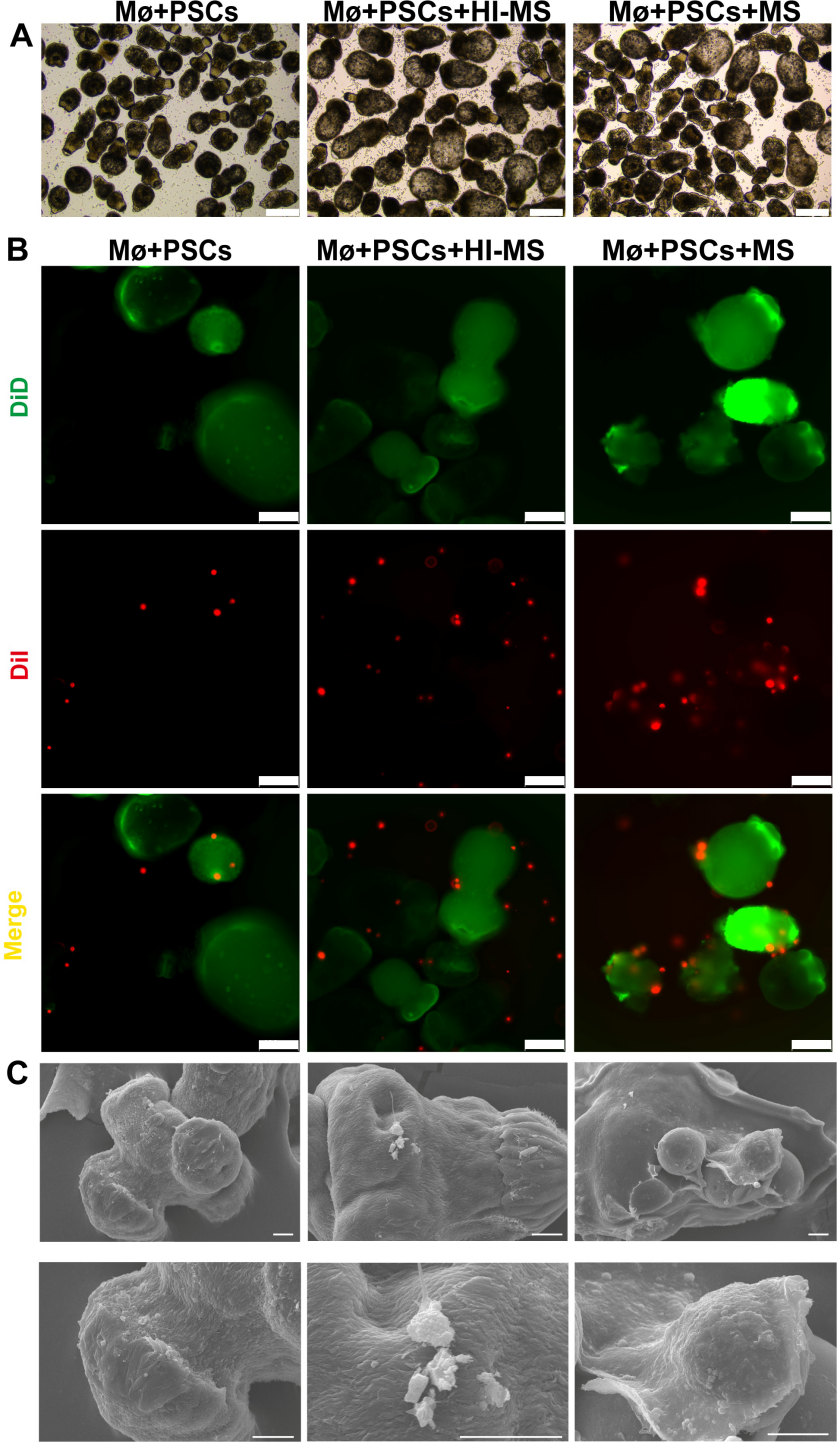
Macrophages eliminate PSCs in a contact-dependent way. **(A)** Morphological alterations in PSCs under co-culture conditions at 48 h Scale bars: 100 μm. **(B)** Contact killing of PSCs by macrophages demonstrated by dual-fluorescence labeling. Scale bar, 100 μm. **(C)** Representative SEM images of macrophages adhering to PSCs in co-culture groups. Scale bars: 10 μm (The difference in length is due to the heterogeneity of parasite size). Images are representative of three independent experiments with similar results.

To determine whether macrophages eliminate *E.m* infection through contact-dependent killing, we co-cultured PSCs with mouse peritoneal macrophages *in vitro* (**Figure 4A**). After 72 h, PSCs in the MS group (supplemented with mouse serum) exhibited significantly suppressed viability. Surrounded by attacking macrophages, the dying PSCs showed numerous vacuoles forming around their tegument (**Figure 4B**). In addition, it has been well documented in previous studies that the complement system plays a critical role in facilitating macrophage recognition and elimination of parasites (18–20). To investigate whether complement components are essential for macrophage killing PSCs, we performed co-culture experiments using heat-inactivated mouse serum (in order to deplete of functional complement components). Consistent with our hypothesis, macrophage cytotoxicity was significantly reduced in the Mø+PSCs+HI-MS group (**Figures 4C**, **5A**). Similarly, dual fluorescent labeling demonstrated a higher number of DiD-labeled macrophages adhered to DiD-labeled PSCs in the Mø+PSCs+MS group compared to the Mø+PSCs+HI-MS group (**Figures 5B**, **Supplementary Figure 1A**). In order to show more clearly how macrophages function to kill PSCs through direct contact, we performed scanning electron microscopy (SEM) on PSCs under different co-culture conditions. In both the Mø+PSCs+MS and Mø+PSCs+HI-MS groups, macrophages were observed firmly attached to parasites, whereas no macrophage adhesion was observed in the Mø+PSCs group. Critically, the Mø+PSCs+MS group exhibited a significantly higher number of adherent macrophages, and these cells displayed typical phagocyte-like morphology (**Figure 5C**).

### 3.5 Complement-dependent trogocytosis mediates macrophage killing of PSCs

**Figure 6:**
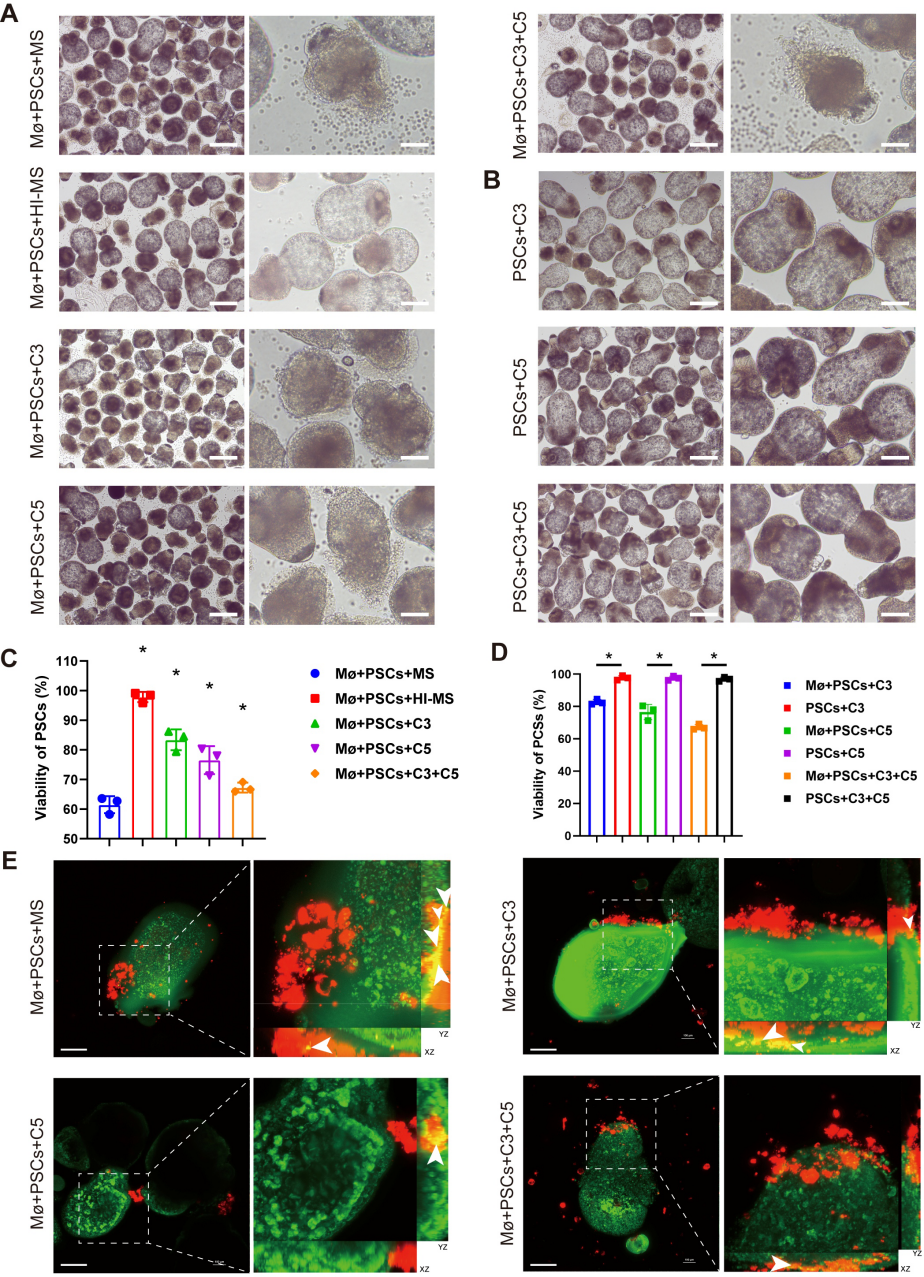
Macrophages eliminate PSCs via trogocytosis in a complement-dependent manner. **(A)** Morphological alterations in PSCs under different co-culture conditions at 48 h in the presence of macrophages. Scale bars: left panels, 100 μm; right panels, 50 μm. **(B)** Morphological alterations in PSCs under different co-culture conditions at 48 h in the absence of macrophages. Scale bars: left panels, 100 μm; right panels, 50 μm. **(C)** Viability of PSCs measured by methylene blue staining under different co-culture conditions. **(D)** Complement toxicity effects were assessed by evaluating the viability of PSCs using methylene blue staining. **(E)** Combined 3D imaging and dual-fluorescent labeling confirmed the trogocytosis of macrophages toward PSCs. The larger images middle are shown as a volume view at a 3.0 zoom factor, while the images on the right and below are shown as slices to visualize the inside of the macrophages, representing YZ-axis and XZ-axis, respectively. Scale bars: 25 μm. White arrows indicate the trogocytosed macrophages. Data and images are presented as mean ± SD from three independent experiments.

To exclude nonspecific interference from other components in mouse serum, we performed rescue experiments with purified recombinant complement proteins (C3 and C5). Co-culture results demonstrated that groups receiving either C3 or C5 supplementation alone, as well as those receiving combined C3 and C5 supplementation, were able to activate macrophage killing activity against PSCs (**Figures 6A, C**). To determine whether the death of PSCs was caused by direct complement-mediated killing (e.g., via membrane attack complex formation), the corresponding toxicity controls we established ruled out this possibility (**Figures 6B, D**). Similarly, we also confirmed that the clearance of PSCs depends on the co-presence of macrophages and mouse serum (**Supplementary Figures 1B, C**). Given that macrophages utilize various contact-dependent mechanisms such as ADCC and perforin/granzyme release, we employed 3D confocal imaging to specifically investigate a potential role for trogocytosis. The imaging confirmed that macrophages adhered to PSCs engulfed PSC membrane components, a hallmark of trogocytosis (**Figure 6E**). Collectively, these results demonstrate that macrophages eliminate *E.m* infection at the early stages via trogocytosis. Moreover, mere contact is insufficient for macrophage-mediated killing. This process requires certain complement components in mouse serum, highlighting the necessity of the *in vivo* microenvironment for effective anti-parasitic immunity.

## 4 Discussion

Alveolar echinococcosis (AE), caused by *E.m* infection, is a lethal zoonotic disease characterized by aggressive liver injury resembling malignancy (21). The early establishment phase is a critical window in which host immunity either eradicates the parasite or permits irreversible cyst formation, a determinant of clinical outcome (22). Here, we demonstrate that macrophage trogocytosis, orchestrated by the complement system, constitutes the dominant mechanism for eliminating *E.m* infection during this decisive phase. Most importantly, the ability of macrophages to effectively invade the interior of the lesion directly determines the fate of the infection: complete parasite clearance in “Regressive Lesions” versus metamorphosis into metacestode vesicles in “Progressive Lesions”.

Macrophages, as central effector cells of innate immunity, play a crucial role in anti-parasitic defense through multiple mechanisms, including phagocytosis of pathogens, secretion of pro-inflammatory cytokines (23–25), and direct killing of parasites via reactive oxygen/nitrogen (26–28), as well as bridging adaptive immunity as antigen-presenting cells (29, 30). On the other hand, parasites employ various strategies to hijack macrophages for immune evasion and long-term survival. *T. gondii* injecting the GRA28 protein to induce macrophage disguise as dendritic cells for migratory dissemination (31). *Trichinella spiralis* cystatin driving M2 anti-inflammatory polarization via the PD-1/STAT6 pathway (32). *E.m* inhibits NF-κB activation in macrophages by chelating calcium ions with phytate to dampen inflammation (33). Thus, elucidating macrophage-parasite immune interactions is critical for developing therapeutic strategies to enhance host immunity and counter parasite immune hijacking. To visually demonstrate how macrophages eliminate parasites at early stage of *E.m* infection, we innovatively employed the lipophilic dye Dil to fluorescently label PSCs, enabling clear *in vivo* tracking. Combined with macrophage-specific fluorescence staining, we revealed that macrophages clear PSCs via invasive direct contact. Notably, using this tracing method, we identified two distinct lesion outcomes: “Progressive Lesions” vs. “Regressive Lesions”, and uncovered residual parasitic components in Regressive Lesions—a detail difficult to discern with conventional histopathology. We propose that lesion typing, independent of traditional metrics like lesion size or cyst weight, could serve as a novel, quantifiable criterion for evaluating *E.m* infection outcomes, offering a scientific basis for drug development and mechanistic studies.

Trogocytosis is a cell-to-cell interaction process in which one cell (the “nibbler”) actively bites off and ingests fragments of another cell’s components through direct contact, leading to target cell damage or death. Unlike classical phagocytosis, trogocytosis involves the removal of small membrane portions without immediate whole-cell engulfment, making it particularly suited for attacking large, multicellular pathogens that cannot be phagocytosed in their entirety (14, 34). A previous study demonstrated that macrophages eliminate *Schistosoma mansoni* in the non-susceptible rodent host Microtus fortis via trogocytosis, establishing this mechanism as a critical determinant of host resistance to parasitic infection (35). Furthermore, in *Entamoeba histolytica*, its pathogenic mechanism is closely associated with trogocytosis. Interestingly, studies indicate that this protozoan directly kills host cells through trogocytosis, causing tissue damage, triggering amebiasis (36, 37). This suggests that trogocytosis may not merely represent a unidirectional host defense against parasites. The present study confirms that macrophages eliminate PSCs through complement-dependent trogocytosis. Using dual-fluorescence labeling and 3D confocal imaging, we observed macrophages actively nibbling fragments from the parasite tegument, a process that was abrogated by heat inactivation of serum complement and restored by supplementation with recombinant C3 or C5. The identification of trogocytosis as a key effector mechanism against *E.m* opens new avenues for therapeutic intervention.

The complement pathway orchestrates macrophage anti-parasitic defense through tripartite mechanisms: opsonization (C3b/C4b deposition enabling CR3-mediated phagocytosis/trogocytosis), inflammatory recruitment (C5a-directed chemotaxis) (38), and direct cytolytic pressure (MAC formation) (39), with CR3 being the master regulator of macrophage trogocytosis, which synergistically activates Syk/PI3K with FcγR signaling and Ca^2+^-dependent NFAT pathway, which drives actin remodeling to form phagocytic synapses, thereby enhancing membrane “nibbling” capacity (40). Conversely, parasites subvert this axis by hijacking complement regulators such as calcitonin or disrupting C3b deposition, thereby impairing trogocytosis, as evidenced by schistosome evasion in CR3-deficient models (41). Consistent with the above studies, our study demonstrated that macrophages exhibited the strongest killing effect on PSCs in co-culture systems containing mouse serum, with trogocytic macrophages displaying characteristic phagocytic-like morphology. Notably, heat inactivation of serum components, which depletes complement factors, significantly attenuated this killing effect. Complement rescue assays further demonstrated that this cytotoxic effect is specifically mediated by a complement-dependent mechanism. Confocal 3D imaging results provide further evidence that this killing mechanism is trogocytosis.

Although this study demonstrates that macrophages contribute to early clearance of *E.m* infection through trogocytosis, significant unresolved questions require further investigation. Our *in vivo* parasite tracing was limited to a 10-day observation period. While this validates the short-term efficacy of the tracer, an extended period is essential to determine the stability of the fluorescent dye *in vivo*. A robust long-term tracking system would fundamentally advance the understanding of parasite-host interactions. For instance, in the study of *Echinococcus*, many research groups have demonstrated that the laminated layer can secrete or shed immunomodulatory parasite-derived components (33, 42–45). However, it is still controversial whether the laminated layer originates from parasite-secreted components or from the host’s own fibrotic response, which could be clarified by continuous *in vivo* monitoring. Additionally, in this study, our evaluation of complement’s role on the trogocytosis effect of macrophages depended solely on the classical heat-inactivation method. This approach cannot exclude potential confounding effects from other mouse serum components, and this limitation was exacerbated by the co-presence of trogocytic and non-trogocytic macrophages in the mouse serum-treated groups, which prevented their effective isolation for deep analysis. Future work should incorporate high-throughput sequencing or proteomics based on trying to obtain purified trogocytic macrophages to elucidate their biological changes and potential signaling mechanisms. Most notably, we observed a perplexing divergence in infection outcomes within individual mice: despite ostensibly uniform physiological conditions, early macrophage-mediated parasite clearance generated two distinct lesion types, the Progressive Lesions and Regressive Lesions. This paradox necessitates resolution of fundamental questions about whether spatial heterogeneity in macrophage-to-parasite ratios, differential intrinsic viability of parasites occupying distinct lesions, or localized immune microenvironment variations drive these dichotomous pathological outcomes. After all, while our findings highlight a pivotal role for macrophages in early parasite clearance, the *in vivo* microenvironment likely involves a coordinated response from multiple innate immune cells. The relative contribution of eosinophils, neutrophils, and other granulocytes remains to be systematically evaluated.

## 5 Conclusions

In conclusion, our study has revealed for the first time that macrophages can participate in the early clearance of *E.m* infection by trogocytosis, and that effective clearance at this stage is a critical window of time for the host to successfully defend against *E.m* infection. An in-depth study of this process is expected to discover the key mechanisms of the host immune system against *E.m* infection, thus providing new important breakthroughs to break the immune tolerance phenomenon existing in the late stage of AE patients and enhance the immunological therapeutic efficacy.

## Data Availability

The original contributions presented in the study are included in the article/**Supplementary Material**. Further inquiries can be directed to the corresponding authors.

## Supplementary material

Supplementary Figure 1 The clearance of PSCs depends on both the presence of macrophages and mouse serum. **(A**) Counts of trogocytosed macrophages on PSCs’ surfaces. Data are presented from three independent experiments. For each experiment, 50 PSCs were counted per group. **(B)** Viability of PSCs measured by methylene blue staining under different co-culture conditions. **(C)** Morphological alterations in PSCs under different co-culture conditions at 48 h. Scale bars: upper panels, 100 μm; lower panels, 50 μm.
