# Supplementary figures and images for: The critical window for host defense: macrophage trogocytosis eliminates Echinococcus multilocularis at the early establishment stage
Source: Front Immunol. 2026 Mar 12;17:1734332. doi: 10.3389/fimmu.2026.1734332 (PMC13017266; doi:10.3389/fimmu.2026.1734332)

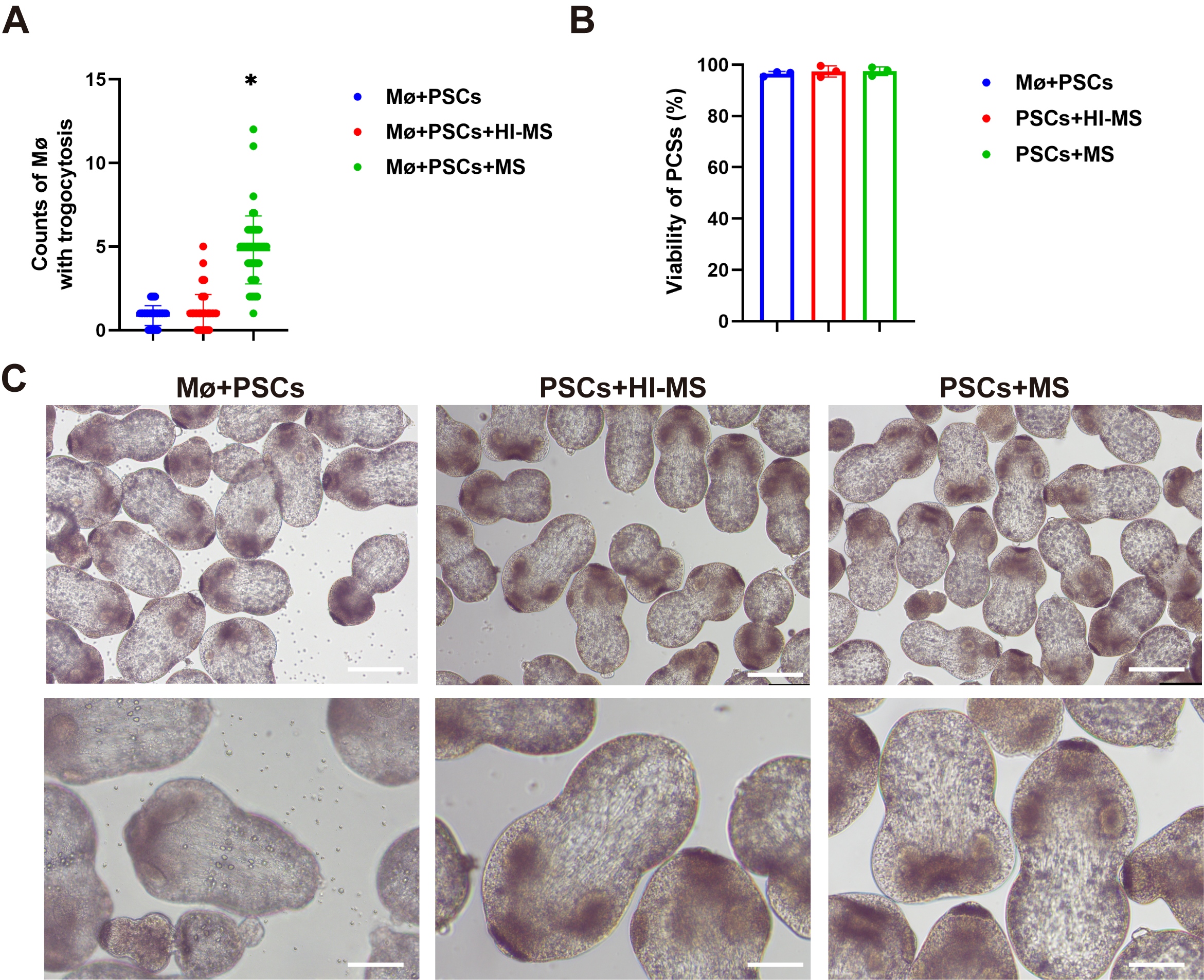

Supplement: Supplementary Figure 1 — The clearance of PSCs depends on both the presence of macrophages and mouse serum. (A) Counts of trogocytosed macrophages on PSCs’ surfaces. Data are presented from three independent experiments. For each experiment, 50 PSCs were counted per group. (B) Viability of PSCs measured by methylene blue staining under different co-culture conditions. (C) Morphological alterations in PSCs under different co-culture conditions at 48 h. Scale bars: upper panels, 100 μm; lower panels, 50 μm. [file Image1.jpeg]
